# Out with the old and in with the new: time to rethink twentieth century chemotaxonomic practices in bacterial taxonomy

**DOI:** 10.1099/ijsem.0.005127

**Published:** 2021-11-30

**Authors:** Peter Vandamme, Iain Sutcliffe

**Affiliations:** ^1^​ Laboratory of Microbiology, Department of Biochemistry and Microbiology, Ghent University, Ghent, Belgium; ^2^​ Northumbria University, Faculty of Health & Life Sciences, Newcastle Upon Tyne, Tyne & Wear, U.K

**Keywords:** bacterial taxonomy, genome sequence, polyphasic taxonomy, chemotaxonomy

## Abstract

Chemotaxonomic methods played an important role in the development of the polyphasic approach to classification of *Archaea* and *Bacteria*. However, we here argue that routine application of these methods is unnecessary in an era when genomic data are available and sufficient for species delineation. Thus, authors who choose not to utilize such methods should not be forced to do so during the peer review and editorial handling of manuscripts describing novel species. Instead, we argue that chemotaxonomy will thrive if improved analytical methods are introduced and deployed, primarily by specialist laboratories, in studies at taxonomic levels above the characterisation of novel species.

Amid impressive progress in microbiome and ecological research, prokaryotic systematics appears increasingly isolated from mainstream microbiology and arguably resistant to innovation [1–3]. It is part of the tradition of gentle self-mockery among taxonomists to state that millennia will be required if we keep on describing novel bacterial species the way we do it now [4, 5]. Nevertheless, change is evidently needed as, to date, fewer than 20 000 prokaryotic species names have been validly published, and millions of species need naming [5]; whilst there is no need to be able to name every single bacterial species, it would be highly desirable to name a far more representative selection. Even though a major proportion of environmental micro-organisms remain as yet uncultivated [6], it is apparent that we already have straightforward cheap procedures sufficient to isolate numerous novel micro-organisms, whilst novel strategies are emerging [7]. Today, there is a tremendous interest in microbiome diversity and function, and there is a general lack of reference cultures for taxa which all too often are likely to be cultivable, as well illustrated by the success of ‘culturomics’ approaches [8]. Large numbers of potentially important bacteria remain to be formally classified with reference strains made available. For example, a recent *Nature Biotechnology* study [9] highlighted that over 60 % of 4644 prokaryotic species in the human gut microbiome represented novel species that lacked representation in international reference databases. The same study reported that for >80 % of the prokaryotic species in the human gut microbiome there were no cultivated isolates. The adoption and further development of high throughput cultivation methods [7, 8] should help systematists work at an increased scale but impediments will remain unless the taxonomic community also adopts new approaches to the publishing of such studies [5]. For instance, in a recent study, Qi *et al*. analysed about 3400 forest soil isolates which were dereplicated into 258 MALDI-TOF mass spectrometry-defined independent strains [10]. While subsequent publications reported three novel *Pedobacter* and six novel *Paenibacillus* species [11, 12], near full length 16S rRNA sequence analyses revealed dozens of additional novel species and genera in this collection [10]. A major reason why only nine novel species from this collection have been formally named so far is the frequently encountered insistence of journal editors that authors provide ‘polyphasic’ taxonomic descriptions that are as comprehensive as possible and that include multiple types of chemotaxonomic data, reflecting recommendations from a decade ago [13]. Here we argue that this latter, specialized, subset of the phenotypic data that can be collected on novel strains (such as fatty acid, polar lipid, polyamine, carbohydrate, quinone and peptidoglycan composition data) is now largely redundant in routine species descriptions.

In contrast to nomenclature, the classification and characterization of prokaryotes is an area that is not formally regulated by the International Code of Nomenclature of Prokaryotes [14], Principle 1.4 of which protects ‘freedom of taxonomic thought or action’. The use of polyphasic analyses in bacterial taxonomy was advocated in the 1990s [15] because, some 25 years ago, it was felt that an integrated approach was the best way to approximate the genomic information that bacteria carry, and so a proposed classification should be the best possible synthesis of as much information as possible. Since then, this has become the standardized approach to microbial taxonomy [13]. However, with the new millennium, bacterial genome sequencing gradually became more affordable and is now established in taxonomic practice [16, 17].

In essence, this is why we have argued against the strict adherence to a rigid definition of polyphasic taxonomy in the twenty-first century and in favour of a revitalization of bacterial taxonomy through an emphasis on genomics [1–3, 5]. While Tindall *et al.* [13] intended to review the key elements to be considered when prokaryotes are characterized, ‘with a view to providing an overview of some of the pitfalls commonly encountered in taxonomic papers’, their paper has subsequently been used as a ‘carved in stone’-checklist for ‘go/no-go’ editorial decisions, thus turning a milestone paper into a millstone around taxonomists’ necks. In particular, the entreatment by Tindall *et al.* [13] that strains ‘should be characterized as comprehensively as possible’ is arguably invalidated by the vastness of the microbial world; put somewhat bluntly, not all prokaryotic species will be of such individual interest as to warrant ‘comprehensive’ characterisation at the time of description, especially those joining the large number of genera with ≥10 members already (currently 340, approaching 10 % of the genera with validly published names; https://lpsn.dsmz.de/text/largest-genera, accessed 30/09/2021). A more pragmatic recommendation is that many strains should be characterized ‘enough’ to allow their discrimination from related taxa and future identification (a return to ‘diagnosis’ rather than ‘description’ [18]). Then strains deemed of interest (for example, following interrogation of their genome sequence) can be subsequently revisited for more extensive characterization.

In this context, it is appropriate to reassess the purpose and utility of chemotaxonomic data. While the *International Journal of Systematic and Evolutionary Microbiology* – and other taxonomic journals – does not have an explicit policy mandating the inclusion of chemotaxonomic data (www.microbiologyresearch.org/journal/ijsem/scope) in novel species descriptions, editors and peer reviewers in daily practice often behave differently. Authors clearly do not have freedom to include only those technical approaches which they deem taxonomically useful and sufficient for their descriptions. Part of this problem is that peer reviewers of taxonomic papers appear unfamiliar with the rules of prokaryotic nomenclature (notably Principle 1.4 quoted above) and that editors of these journals likewise do not correct inappropriate requests such as ‘it is *obligatory* to do fatty acid, carbohydrate, quinone and peptidoglycan analyses when you report a novel species’, ‘it is *obligatory* to do DNA fingerprinting when reporting a novel species’ or ‘you *must* provide more important chemotaxonomic data, such as peptidoglycan type, whole-cell sugars, polar lipids, respiratory quinone and cellular fatty acids’ (all were copied from recent decision letters, italics added for emphasis).

Despite a longstanding concern that ‘few scientists now have extensive experience’ (i.e. the requisite skills) for the ‘interpretation of chemotaxonomic data’ [13], editors still insist on authors providing multiple types of chemotaxonomic analyses that have, in bacterial taxonomy, essentially descriptive value only. In the classic taxonomic trinity of classification, identification and nomenclature [19], the practical value of chemotaxonomic data today is virtually non-existent for species delineation, when compared to the taxonomic resolution of phylogenomic methods. Mostly, what is confirmed by traditional chemotaxonomy is that the properties of a new member of genus X are very similar/identical to those of known members of genus X (information that could have been confidently predicted from the phylogenetic placement of the new species in genus X!). Indeed, in some cases, the phylogenomic approach is needed to resolve ambiguities introduced by chemotaxonomy [20]. Moreover, chemotaxonomic data is often laborious to acquire (typically requiring some combination of extraction, purification, derivatization and analysis of cellular constituents) and thus relatively expensive, diverting scarce resources from more informative work. This expense is compounded by the need for parallel testing of novel strains and references strains in order to compensate for the fact that several standard chemotaxonomic characters are notably affected by changes in growth conditions, thus undermining the ability to directly compare data from the literature and demanding stringent intra-laboratory standardization, which is rarely documented. Inter-laboratory standardization is yet more difficult still and the ‘manual’ nature of many traditional methods has resisted the introduction of automation and the accompanying benefits of robust quality control processes.

Whilst chemotaxonomic data can be of value, it is clearly better employed in studies at taxonomic ranks above the species level. Knowledge of cellular components such as lipids and cell walls may be, or become, valuable with increasing insights into – for instance – microbe–host interactions. However, the twentieth century analytical tools employed by many taxonomists need to be replaced in such studies by much higher-resolution technologies to be truly informative. For example, the resolution of two-dimensional thin-layer chromatography, which remains routinely used in taxonomic polar lipid studies, falls far short of contemporary mass spectrometry lipidomic methods, which can resolve the few spots observed (but often not identified) on thin-layer chromatography plates into multiple lipid species (for a very clear example, see the resolution of seven bulk lipid classes into >60 individual lipids by Rashid *et al.* [21]). Similarly, the application of the thin-layer chromatography to sugar profiling of whole-cell hydrolysates remains a crude broad brushstroke approach compared to the resolution of metabolomic fingerprinting. Even fatty acid analyses, which have benefited greatly from the standardization provided by commercial diagnostic systems, still face challenges when comparing datasets between laboratories and over time.

Collaborations with analytical chemists to introduce state-of-art methodologies are thus needed to revitalize the chemotaxonomic toolkit. Indeed, it is possible to consider a future for chemotaxonomy where the application (and re-application, and re-application…) of a checklist of entrenched methodologies in separate single species descriptions is, instead, replaced by comprehensive, informative surveys carried out in specialized laboratories. For example, Schumann *et al.* [22] recently applied what they themselves described as ‘perceived … “occult” methods’ to re-evaluate chemotaxonomic characteristics of three type strains in the family *Ruaniaceae *: reanalysis using state-of-the-art methods allowed correction of the peptidoglycan structural data for two type strains, and clarification of the menaquinone profile of one of them. In addition, they identified ribose as the major whole-cell sugar in three type strains previously reported to contain various sugars other than ribose. Ironically, such ‘synoptic’ studies were more common when chemotaxonomy was first introduced in the 1970s: it is time to revive the practice (as we argued more than a decade ago [23]). Such studies would renew appreciation of the utility of appropriately selected chemotaxonomic data, particularly at taxonomic ranks above the species. Moreover, collaboration between bioinformaticians and chemotaxonomists should allow the further development of biosynthetic/metabolic pathway analyses into ‘*in silico* chemotaxonomy’ approaches [20, 23, 24]. Further development of spectral fingerprinting methods should also help overcome an intrinsic lack of ‘portability’ of data acquired from twentieth century chromatographic techniques and are likely to involve safer, ‘greener’ protocols than the often hazardous older methods. In this regard, it is notable that the one type of chemotaxonomic information that has practical value in daily diagnostic microbiology today is MALDI-TOF mass spectrometry analysis [25]. Novel species that have a MALDI-TOF mass spectrometry profile that is different from that of their nearest neighbours can be recognized by countless microbiologists who use MALDI-TOF mass spectrometry technology on a daily basis for diagnostic purposes by simply adding a reference spectrum to their mass spectrum library. Undoubtedly, it would significantly benefit the systematics community and further drive the adoption of this technology if freely accessible and iterative spectral database(s) were made available.

The common practice of single-strain-species descriptions resulted in an ‘assembly line’ of formulaic papers [26], a situation reinforced by peer reviewers and/or editors who propagate the same dogmatic adherence to the formula [1, 5]. Traditional taxonomic journals have started to change gears and discourage publication of single-strain-species descriptions [27]. Today, editors request authors to functionally explore genome sequences or to demonstrate novel biology and features of interest in a proposed novel taxon [28]. We urge editors of taxonomic journals (and peer reviewers) to take the next step. A genome sequence suffices to prove that a strain represents a novel species or not. The request, as a *conditio sine qua non*, to generate multiple types of chemotaxonomic information as part of a novel species description imposes a burden that is unnecessarily high and counterproductive. It may be naive to strive to formally name all prokaryotic species, but many fields are in dire need of reference cultures that correspond to sequences that have been detected through cultivation independent analyses. We argue that a genome sequence analysis, a basic characterization of growth characteristics and the public deposit of type strains should suffice for proposing novel species. Beyond this, authors should have freedom to include (extensive) phenotypic and chemotaxonomic testing, or not, as they deem appropriate.
